# Genome Organization and Comparative Evolutionary Mitochondriomics of Brown Planthopper, *Nilaparvata lugens* Biotype 4 Using Next Generation Sequencing (NGS)

**DOI:** 10.3390/life12091289

**Published:** 2022-08-23

**Authors:** Guru-Pirasanna-Pandi Govindharaj, Soumya Bharti Babu, Jaipal Singh Choudhary, Muhammad Asad, Parameswaran Chidambaranathan, Basana-Gowda Gadratagi, Prakash Chandra Rath, Naiyar Naaz, Mariusz Jaremko, Kamal Ahmad Qureshi, Uttam Kumar

**Affiliations:** 1 Division of Crop Protection, ICAR-National Rice Research Institute, Cuttack 753006, India; 2 ICAR-Research Complex for Eastern Region, Farming System Research Centre for Hill and Plateau Region, Ranchi 834010, India; 3 College of Life Science, Fujian Agriculture and Forestry University, Fuzhou 350002, China; 4 Smart-Health Initiative (SHI) and Red Sea Research Center (RSRC), Division of Biological and Environmental Sciences and Engineering (BESE), King Abdullah University of Science and Technology (KAUST), Thuwal 23955, Saudi Arabia; 5 Department of Pharmaceutics, Unaizah College of Pharmacy, Qassim University, Unaizah 51911, Saudi Arabia; 6 College of Plant Protection, Fujian Agriculture and Forestry University, Fuzhou 350002, China

**Keywords:** phylogeny, phloem feeder, sucking pest, mitochondria, NGS

## Abstract

*Nilaparvata lugens* is the main rice pest in India. Until now, the Indian *N. lugens* mitochondrial genome has not been sequenced, which is a very important basis for population genetics and phylogenetic evolution studies. An attempt was made to sequence two examples of the whole mitochondrial genome of *N. lugens* biotype 4 from the Indian population for the first time. The mitogenomes of *N. lugens* are 16,072 and 16,081 bp long with 77.50% and 77.45% A + T contents, respectively, for both of the samples. The mitochondrial genome of *N. lugens* contains 37 genes, including 13 protein-coding genes (PCGs) (*cox1*-3, *atp6*, *atp8*, *nad1-6*, *nad4l*, and *cob*), 22 transfer RNA genes, and two ribosomal RNA (*rrnS* and *rrnL*) subunits genes, which are typical of metazoan mitogenomes. However, both samples of *N. lugens* mitogenome in the present study retained one extra copy of the trnC gene. Additionally, we also found 93 bp lengths for the *atp8* gene in both of the samples, which were 60–70 bp less than that of the other sequenced mitogenomes of hemipteran insects. The phylogenetic analysis of the 19 delphacids mitogenome dataset yielded two identical topologies when rooted with *Ugyops* sp. in one clade, and the remaining species formed another clade with *P. maidis* and *M. muiri* being sisters to the remaining species. Further, the genus *Nilaparvata* formed a separate subclade with the other genera (*Sogatella*, *Laodelphax*, *Changeondelphax*, and *Unkanodes*) of Delphacidae. Additionally, the relationship among the biotypes of *N. lugens* was recovered as the present study samples (biotype-4) were separated from the three biotypes reported earlier. The present study provides the reference mitogenome for *N. lugens* biotype 4 that may be utilized for biotype differentiation and molecular-aspect-based future studies of *N. lugens*.

## 1. Introduction

The brown planthopper, *Nilaparvata lugens* (Stål.), imposes a serious threat to rice cultivation all over the world. It is a monophagous pest that attacks rice crops from the early vegetativeto the late grain-maturation stage [1,2]. Infested plants have typical hopper burn symptoms and smaller panicles with fewer filled grains [3]. In view of its economic damage prospects, it is considered to be one of the disastrous pest species of rice in tropical, subtropical, and temperate regions [3]. *N. lugens* are thermophilic in nature and have a tendency for long-distance migration. The characteristics of its quick proliferation combined with a strong migration ability make this pest the most dreaded pest in the Indian subcontinent, where it causes severe yield losses, and in some places, even the total failure of the crop has been recorded [2,4]. *N. lugens* biotype 4 is the most ravaging one among the four biotypes reported globally and is the predominant pest of rice in most Asian nations [5].

In general, an insect species’ mitochondrial genome usually encrypts a set of 13 protein-coding genes (PCGs), 22 transfer RNAs (tRNAs), and two ribosomal RNAs (rRNAs) in a concise double-standard closed circular DNA that falls approximately in the range of 14 to 40 Kbp [6]. The mitochondrial genome typically comprises a unique non-coding section termed the control region (CR), which possesses more A + T nucleotides [7,8,9] and sections for genome replication and the commencement of gene transcription [6,10]. Reports suggest that mitogenome content and organization were widely employed in the fields of evolutionary and comparative genomics, population genetics, phylogenetics, and molecular evolution [9,11].

To date, out of the 2200 reported species from Delphacidae, merely 17 mitogenomes species representing 11 species are available in the National Center for Biotechnology Information (NCBI) GenBank, including *N. lugens*, *N. muiri*, *N. bakeri*, *Changeodelphax velitchkovskyi*, *Laodelphax striatella*, *Matsumuramata muiri*, *Peregrinus maidis*, *Sogatella frucifera*, *S. kolophon*, *Ugyops* sp, and *Unkanodes sapporona*. This suggests that more research is needed before finalizing the evolution theories in this family, which belongs to the order Hemiptera, suborder Auchenorrhyncha. Various theories have been proposed with respect to suborder Auchenorrhyncha, which at present constitutes both Fulgoromorpha and Cicadomorpha. Earlier researchers suggested different classification patterns with the following theories (1) Auchenorrhyncha was designated as monophyletic and assigned as a sister cluster to Heteropterodea (Heteroptera + Coleorrhyncha) [12], (2) monophyletic and assigned as a putative sister cluster to Sternorrhyncha [13], (3) non-monophyletic and both Cicadomorpha and Fulgoromorpha were assigned in separate phylogenetic positions within Hemiptera [14]. This debate is still unclear, and recent studies support both monophyletic [15] and non-monophyletic [16] theories; thus, more evidence, such as the various members’ mitogenomes, is required to resolve the problem in Fulgoromorpha. Fulgoromorpha needs further evaluation to attain a clear phylogenetic relationship and a consequential classification method [17].

Therefore, in the present investigation, we assembled two mitochondrial genomes of *N. lugens* biotype 4 with next-generation sequencing (NGS) to offer fresh evidence regarding the Indian population of *N. lugens*. The present study reports the mitochondrial genome of *N. lugens* biotype 4 from India, comprising gene assembly, gene overlaps, tRNA secondary structure, codon usage, nucleotide composition, and the non-coding control region. The genome organization was compared with the available complete mitogenomes of other delphacids viz. *N. lugens*, *N. muiri*, *N. bakeri*, *Changeodelphax velitchkovskyi*, *Laodelphax striatella*, *Matsumuramata muiri*, *Peregrinus maidis*, *Sogatella frucifera*, *S. kolophon*, *Ugyops* sp., and *Unkanodes sapporona.* The sequences of the present study, along with the available mitogenome sequences of infra-order Fulgomorpha in the NCBI database, were used, and the phylogeny of Fulgomorpha was revamped. The present study also provides the reference sequence of the mitogenome for *N. lugens* biotype 4 that may be utilized for biotype differentiation and molecular-aspect-based future studies of *N. lugens*.

## 2. Materials and Methods

### 2.1. Mitogenome Sequencing and Assembly

The *N. lugens* populations were reared in a glass house at 28 ± 2 °C, 70 ± 5% relative humidity, and a 14:10 h (light:dark) photoperiod at the ICAR-National Rice Research Institute, Cuttack, India. The glasshouse was equipped with an air-cooling and lighting system to maintain an optimum range of temperature, relative humidity, and photoperiod. The initial culture was collected from the infested rice plants at the institute’s research field and released on the susceptible variety TN1. The collected insects were identified based on their morphological description according to Wilson (1991) [18].

The mitochondrial DNA was extracted from two samples of female hoppers (40 mg hoppers per sample and 10 individuals were taken for each sample) using the Abcam Mitochondrial DNA Isolation Kit (ab65321) according to the manufacturer’s protocol (Abcam, Cambridge, UK). Xcelris Labs Limited in Ahmedabad, India, performed mitogenome sequencing and Illumina library preparation. Following the manufacturer’s instructions, a paired-end sequencing library was created using the Truseq Nano DNA library preparation kit. The library preparation process began with 100 ng of mtDNA fragmentation, followed by end adapter ligation. The ligated products were amplified using PCR to achieve a size range of 200–1000 bp. To ensure maximum yields from limited amounts of starting material, a high-fidelity amplification step was performed using HiFi PCR Master Mix. The amplified library was analyzed using an Agilent Bioanalyzer 2100 with a High Sensitivity DNA chip. The sequencing was undertaken with an Illumina MiSeq Sequencing System (Illumia Inc., San Diego, CA, USA) and a 2 × 150 bp chemical kit. The library was normalized to the concentration of 16 nM after finding the mean peak size from the bioanalyzer profile and library Qubit concentration and subsequently diluted to the concentration of 20 pM. MiSeq was used to sequence the template fragments in both the forward and reverse directions. On paired-end flow cells, the samples were bound to complementary adapter oligos using kit reagents. The adapters were created to allow selective cleavage of the forward strands after the reverse strand was re-synthesized through sequencing. The raw reads were assembled and proofread into the CLC genomics workbench program version 6 v.2.5.3 (http://www.clcbio.com, accessed on 4 April 2021). The FASTX-Tool Kit was used to trim and preprocess the Fastq file (http://hannonlab.cshl.edu/fastx_toolkit/, accessed on 4 April 2021). The high-quality reads were assembled using NOVOPlasty 4.2. The blastn algorithm was used to identify contigs containing mitogenome sequences in the NCBI database (non-redundant nucleotide).

### 2.2. N. lugens Mitogenome Annotation

The MITOS web server was employed for the prediction of the all the mitochondrial gene coding regions, viz., protein-coding genes (PCGs), 22 transfer RNAs (tRNAs), and 2 ribosomal RNAs (rRNAs) by keeping the invertebrate mitochondrial genetic code as the base [19]. Additionally, all of the PCGs and rRNAs genes were physically determined by a comparison to the 17 mitogenomes of the 11 other species collected from GenBank, namely *N. lugens* (two samples from the present study; JN563995-97; KC333653-54; JX880069; MK606371), *N. muiri* (JN563998), *N. bakeri* (KC333655), *Changeodelphax velitchkovskyi* (MG049916), *Laodelphax striatella* (JX880068), *Matsumuramata muiri* (MW288929), *Peregrinus maidis* (MG049917), *Sogatella frucifera* (MK907866), *S. kolophon* (MW009064), *Ugyops* sp (MH352481), and *Unkanodes sapporona* (MN544774). The CG view server, ClustalW, and DNA mfold server with default parameters were used for the construction of circular mitochondrial maps, alignment, and secondary structure folding, respectively, according to the standard procedure [20,21,22]. The Mito/Chloroplast model was used to recheck the two-dimensional tRNA structures downloaded from MITOS using tRNA scanSEv 1.21 with a covariance cut-off value of 15.0 [22].The composition of the skew analysis was calculated using formulae GC skew = [G−C]/[G+C] and AT skew = [A−T]/[A+T] [23]. The intergenic spacers between the genes and the overlapping regions were calculated manually. DnaSP 5.0 software (Universitat de Barcelona, Barcelona, Spain) was used for the calculation of the non-synonymous substitution rate (Ka), and the synonymous substitution rate (Ks) with Jukes-Cantor adjusted Ka/Ks (JKa/JKs) for each PCG [24]. The whole assembled and annotated *N. lugens* mitogenome was submitted to the NCBI GenBank with accession numbers: OK585089 and OM372598. The final sequence read achieves (SRAs) were deposited in the NCBI SRA database under the Bioproject: PRJNA841994 and Biosamples accession numbers: SAMN28637499 and SRX15441812, available at https://www.ncbi.nlm.nih.gov/Traces/study/?acc=PRJNA841994 (accessed on 6 July 2022).

### 2.3. Phylogenetic Analysis

All of the available complete mitogenomes of the members of Delphacidae (19 mitogenomes) were selected as an in-group, and two species of Cicadamorpha and three species of heteroptera were used as outgroups for the phylogenetic analysis. Out of the 19 mitogenomes of Delphacidae, two samples were sequenced in the present study, and the remaining samples of Delphacidae were collected from the NCBI database.

Concatenated nucleotide sequences of 13 PCGs datasets were used for the phylogenetic analysis. MAFFT 7 (https://ma_t.cbrc.jp/alignment/server/, accessed on 16 July 2022) (Osaka University, Osaka, Japan)was used to align the PCG on the basis of codons for amino acids [25]. Similarly, to remove the ambiguously aligned sited from the PCG elements, GBlocks v.0.91b (http://molevol.cmima.csic.es/castresana/Gblocks/Gblocks_documentation.html, accessed on 6 July 2022) was used [26]. MEGA 10.0 was used for the final quality checking for all of the alignments. PhyloSuite 1.2.1 was used for the concatenation of all gene alignments [27]. For phylogenetic reconstruction, a PCG123 matrix was created with the thirteen PCGs combined into a single sequence in the following order: *nad2, cox1, cox2, atp8, atp6, cox3, nad3, nad5, nad4, nad4l, nad6, cytb,* and *nad1*. The phylogenetic reconstruction was completed using maximum likelihood (ML) analysis. Partition Finder 2.1.1 with the Bayesian Information Criterion (BIC) and a greedy algorithm (www.phylo.org, accessed on 16 July 2022) was used for selecting the best partitioning schemes [28]. As per BIC, the GTR+I+G model was ideal for analysis with nucleotide alignments. ML analysis was inferred by the PHYML online web server [29]. Nodal support among branches was evaluated by bootstrap resampling calculated using 100 replicates. The constructed phylogenetic tree was visualized with Interactive Tree of Life (iTOL version 4.0) software (Biobyte solutions, Heidelberg, Germany) [30].

## 3. Results and Discussion

### 3.1. Sequencing and Assembly of N. lugens Mitogenome

Recent molecular techniques, such as next-generation sequencing (NGS), have made it easier to understand biological organism evolution, phylogeny, and developmental functions. In comparison to the Sanger method of sequencing, NGS is more efficient at producing a large volume of sequence data in less time [31]. NGS has been widely used for a variety of applications, including bacterial genome sequence [32], the de novo genome assembly of various arthropods, whole-genome assembly, and mitochondrial genome sequencing of living organisms and/or herbarium and museum specimens [33,34,35]. The present study used the genomic DNA libraries to acquire *N. lugens* mitochondrial DNA sequence data. Precisely, a total of 30.4 and 32.5 million reads were acquired from the single-end reads from samples 1 and 2, respectively, on the paired-run Illumina MiSeq sequencing platform of 2 × 150 bp read size. The present assembly of >30 million genomic DNA reads from the library led to a total of 151,745 and 156,328 contigs for Sample1 and Sample2, respectively, which were searched for similarity against NCBI’s Nucleotide (NT) database using the BLASTN algorithm. A total of 1009 scaffolds in Sample1 and 1092 scaffolds in Sample2 showed similarity to the *N. lugens* mitochondrial genome (accession number- LC461185). The study results are such that the mitogenomes of both the samples were highly identical (99.9%) with a negligible variation. Therefore, to maintain the clarity of reading, most of the findings of only one sample are discussed hereafter unless mentioned elsewhere in the manuscript.

### 3.2. N. lugens Genome Organization and Rearrangement

**Figure 1:**
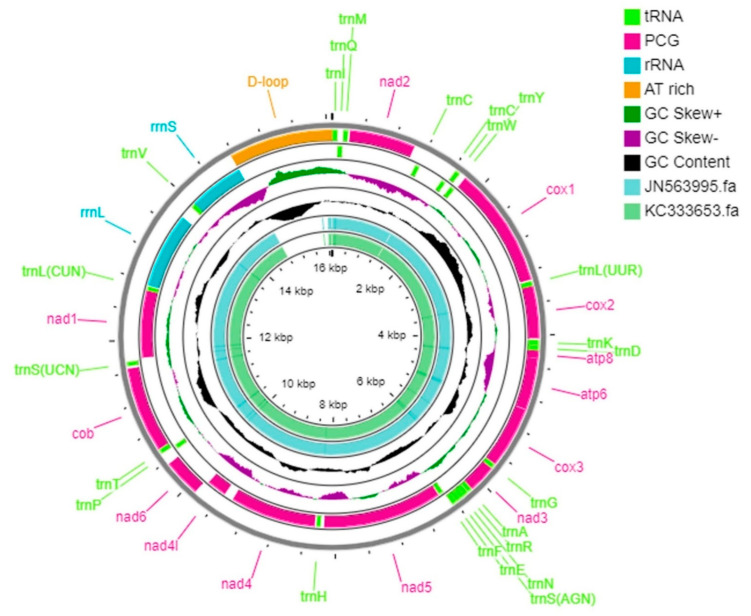
*Nilaparvata lugens* mitochondrial genome map (PCGs, rRNA, tRNAs, and CR) is indicated in the first outer circle. GC content and GC skew are represented in second and third circle, respectively, and inner circles are previously submitted sequences biotype 1 (JN563995) and L (KC333653) genome map.

*N. lugens* whole mitogenome (Genebank accession: OK585089) is a closed-circular particle of 16,072 and 16,081 bp in length that falls in the range of earlier-reported whole *N. lugens* mitogenomes with a length of 14,364 bp (JN563995) to 17,619 bp (JX880069). Both mitochondrial genomes contain the 37 genes found in metazoan mitogenomes [6], which include13 PCGs (*cox1-3*, *atp6*, *atp8*, *nad1-6*, *nad4l*, and *cob*), 22 tRNAs (one for each amino acid, two each for Serine, Leucine, and Cysteine), and two rRNA (*rrnS* and *rrnL*) genes (Figure 1, Supplementary Tables S1 and S2).

**Figure 2:**
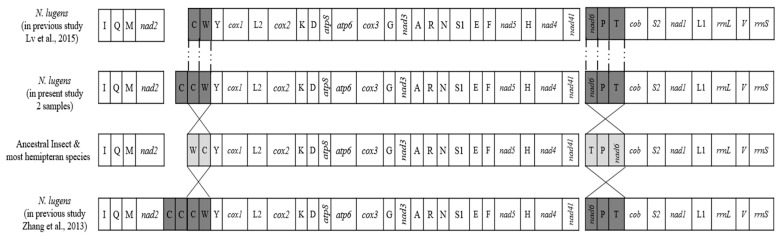
Linearized depiction of the mitogenome gene arrangements in *Nilaparvata lugens* in comparison with putative ancestral arthropod, and *N. lugens* in previous study [27,36]. Shaded boxes indicate genes concerning mitogenome rearrangement.

The mitochondrial gene order of *N. lugens* is significantly different from the mitochondrial gene order of *D. yakuba* (the first insect mitochondrial genome sequenced) and most of the hemipteran species (Figure 2). The present study observed two rearrangement regions in *N. lugens* viz., the first one was *trnC-trnW*, and the other was *nad6-trnP-trnT*, which was similar to Zhang et al. (2013) and Lv et al. (2015) [27,36]. The rearrangement of the *trn* genes was also reported from *Neuroctenus parus* (*trnI* and *trnQ*) and *Physopelta gutta* (*trnT* and *trnP*) [37]. Moreover, the striking feature in the present study was the presence of one additional *trnC* gene (Figure 2) in both the samples of *N. lugens*, which was one less than Zhang et al. (2013), where two additional *trnC* genes in *N. lugens* were reported, but Lv et al. (2015) reported only one *trnC* gene, which tallies to most hemipteran insects sequenced thus far [38]. Indian populations of *N. lugens* representing biotype 4 differ from the other three biotypes by having an extra copy of the *trnC* genes.

The non-coding region, which is a long uninterrupted chain corresponding to the possible homologous of the insect A + T rich region by basic configuration, positional homology, and base content, was present between *rrnS* and *trnI*, analogous to positions 14,769 to 16,072 in Sample 1 and 14,763–16,081 in Sample 2, with a total length of 1304 bp in Sample 1 and 1319 bp in Sample 2 (Figure 1; Supplementary Figure S1). *N. lugens* genome organization was condensed with 1198 and 1193 bp for Sample 1 and Sample 2, respectively, distributed in 27 intergenic spacers ranging from 1 to 345 bp and adjoining genes overlapping by a total of 10 bases ranging from 1 to 6 bp at five boundaries. Except for whitefly (*Trialeurodes vaporarium*), the mitogenome of *N. lugens* was the lengthiest in Hemiptera that has been reported so far [26]. The rare longest length of the mitogenome in *N. lugens* was partially owing to the long putative control region and a long repeat region comprising additional repeats of the *trnC* gene. However, their functional role is unknown; similar gene duplication has also been reported in the mitogenomes of *Metaseiulus occidentalis* [39] and *Leptotrombidium pallidum* [40].

### 3.3. N. lugens Nucleotide Composition and Skewness

Both strand-specific and genome-wide compositional biases were discovered in the base composition. Analogous to the already-reported insect mitogenomes, the nucleotide composition in *N. lugens* was biased towards adenine and thymine with 77.51% (A = 42.6%; T = 34.9, G = 8.8, C = 13.7%) in Sample1 and 77.50% (A = 42.6%; T = 34.8, G = 8.8, C = 13.6%) in Sample2. This bias is well within the range recorded for the other mitochondrial genomes of delphacid species, from 75.73% in *Changeondelphax velitchkovskyi* to 77.75% in *Peregrinus maidis*. The A + T content was independently analyzed for all of the PCGs (76.56% in both), tRNAs (78.49% in both), rRNAs (77.17% in both), and the CR (87.34 in Sample 1 and 87.08% in Sample 2) regions also falls into the earlier-reported range of the complete mitogenomes of the 11 delphacids species analyzed in this experiment. The PCGs of the two strands (J-majority and N-minority) were analyzed separately, and it was found that the J-strand (comprising nine PCGs) had a 75.68% A + T content, which is lower than the N-strand (comprising four PCGs) value of 78.53%. Additionally, a strong bias for T content with 38.59% and 52.23%, respectively, for the J-strand and N-strand in comparison to the corresponding values of 37.08% and 26.30% for A content was observed in the PCGs. Similarities have been observed in earlier reported Hemipeteran mitochondrial genomes and also in other insects [9,41,42,43].

The relative synonymous codon usage (RSCU) and the profusion of the codon families in PCGs in *N. lugens* are summarized in Table 1. The codon usage clearly reflects the nucleotide bias. The analysis showed that theA+T content was significantly higher in the third codon position due to the code degeneracies. Further, the T nucleotide was overrepresented in the second codon position, as previously reported in other insects’ mitochondrial genomes [9,41,44]. Likewise, all the three codons in PCGs on the N-strand have comparatively higher G content than C content, while the reverse pattern was found in the J-strand. This base compositional biasis equivalent to the typical tendency in the mitogenome towards a lower G content [45]. The RSCU of the NNA and NNU codons > 1 directed that the third position of the U/A has a high frequency of codon usage in the *N. lugens* mitochondrial genome (Table 1). Furthermore, *TTA*-*Leu* (3.88), *ATT*-*Ile* (1.76), *ATA*-*Met* (1.72), *AAA*-*Lys* (1.72), *AAT*-*Asn* (1.66), and *TAT*-*Tyr* (1.66) showed a strong bias towards A+T rich codons. The total number of non-stop codons in the two samples of the present study mitogenomes of *N. lugens* are alike, with 3448 bp for both samples. Thus, similar behavior was observed in regard to the codon families among the two samples. The most often used amino acid is Leu (15.01%), followed by Phe (11.32%), Ile (11.16%), Met (7.24%), and Asp (5.09%). The quantity of these five amino acids is >49.84%.

**Figure 3:**
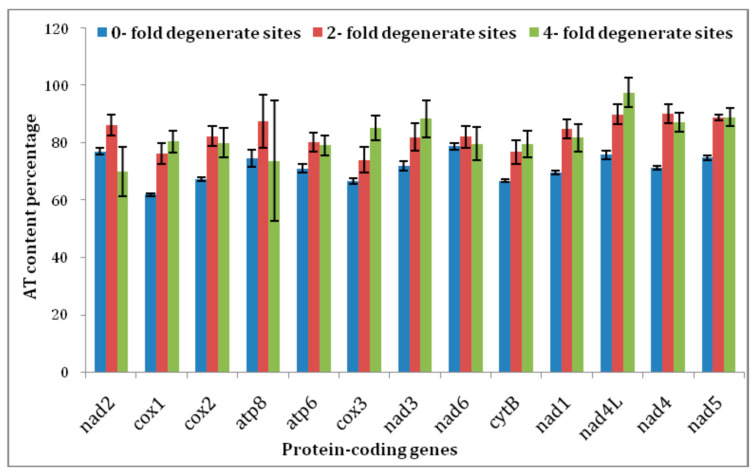
The AT content percentage of 0-fold degenerate sites, 2-fold degenerate sites, and 4-fold degenerate sites in each protein-coding gene of 19 Delphacidae species from this study, including two *Nilaparvata lugens* species. The black line at the top of each bar represents the standard deviation value (SD).

Numerous attempts have been made by earlier researchers on insect mitogenome A + T content of different genes to examine the among-site rate variation (ASRV) and base compositional heterogeneity. Each PCGs A + T content of zero-fold sites (P_OFD_), two-fold (P_2FD_), and four-fold degenerate sites (P_4FD_) was determined for 19 delphacids mitogenome (*N. lugens*; *N. muiri*; *N. bakeri*; *Changeodelphax velitchkovskyi*; *Laodelphax striatella*; *Matsumuramata muiri*; *Peregrinus maidis*; *Sogatella frucifera*; *S. kolophon*; *Ugyops* sp.; *Unkanodes sapporona*) (Figure 3). Further, the present study identified that most of the PCGs showed significantly lower variation in A+T content except for the *nad6* gene at P_OFD_ in comparison to both P_2FD_ and P_4FD_ sites among the 19 delphacid mitogenomes. The A + T content of *nad6* was higher at P_OFD_ (78.8 ± 1.12) in comparison to both P_2FD_ (82.1 ± 3.73) and P_4FD_ (79.7 ± 5.83).

Both AT and GC skew were worked out for all of the available whole mitochondrial genomes of Delphacidae species, displaying that the AT skewness of the *N. lugens* mitochondrial genome was slightly positive (0.099 in Sample 1 and 0.10 in Sample 2), demonstrating the presence of more As than Ts. Earlier reports from Delphacidae species were also similar to our results, with a range of 0.089 in *N. lugens* to 0.288 in *Ugyops* sp. Though the GC skew was negative in all the selected Delphacidae mitochondrial genomes (−0.133 to −0.271), it is noteworthy that the GC skew of *N. lugens* was −0.215 in Sample1 and −0.214 in Sample2, denoting the presence of more Cs than Gs, analogous to the skewness of earlier reported hemipteran, lepidopteran, dipteran, and other Animalia mitochondrial genomes [46,47,48].

### 3.4. Protein-Coding Genes

The mitogenome of *N. lugens* in the present study comprises the complete set of 13 PCGs encoded in the animal mitogenome (Supplementary Tables S1 and S2). Apart from the *nad6* gene, all of the other PCGs were organized along the genome in accordance with the anticipated typical order of insects [6]. Most of the PCGs start with a characteristic ATN initiation codon, i.e., ATA in *nad2*, *nad3*, and *atp6*; ATG in *cox1*, *cox3*, *nad5*, *nad4L*, *cyb*, and *nad1*; ATT in *cox2*, *atp8*, *nad4*,and *nad6*. Among the insects, methionine encoding initiation codons such as ATA or ATG are the most common ones [46,47], whereas the ATT initiation codon is unusual but frequently found in *N. lugens* as well as other Delphacids [27,36,38,49]. Standard TAA termination codons are found in four (*cox1*, *atp6*, *nad4*, and *nad4L*) PCGs. For the *nad2*, *cox2*, and *cox3* genes, *N. lugens* has two different stop codons, TTA for the former and TGA for the latter two genes (Supplementary Tables S1 and S2). The remaining PCG exhibited an incomplete stop codon as TT in nad5 and T in *atp8*, *and3*, *nad6*, *cyptb*, and *nad1*.

In the present study, the length of the *atp8* gene of *N. lugens* was 93bp for both samples, which was 6 bp less than the earlier report [27]. However, the striking feature of the *atp8* gene is that it is the shortest gene in insects known so far and the largest (228 bp) *atp8* gene found in *Bemisia tabaci* [50]. The insect *atp8* genes range from 138 bp in *Pachypsylla venusta* [51] to 183 bp in *Lymantria disper* [52], with the majority falling in or around 160 bp [27]. The present study reported that nearly 70bp of the *atp8* gene sequence were lost in *N. lugens*, and their functional characterization needs to be ascertained for any significant changes occurring in the insect with respect to gene loss.

### 3.5. N. lugens Ribosomal RNA Genes, Transfer RNA Genes and Associated tRNA Structure

**Figure 4:**
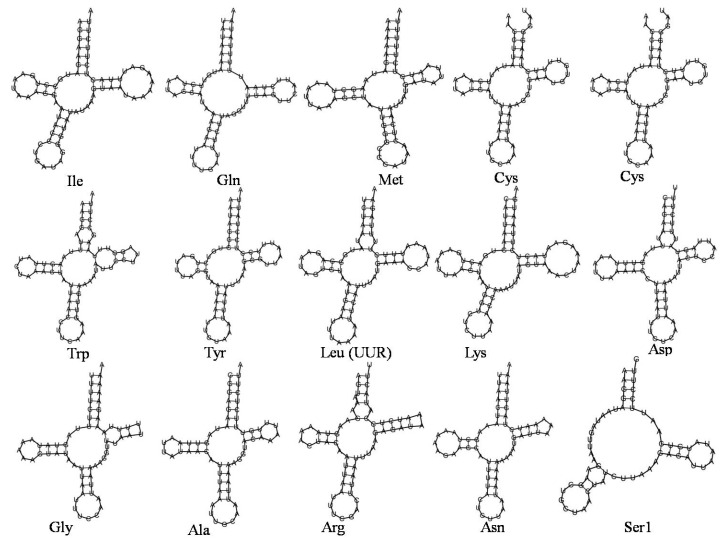

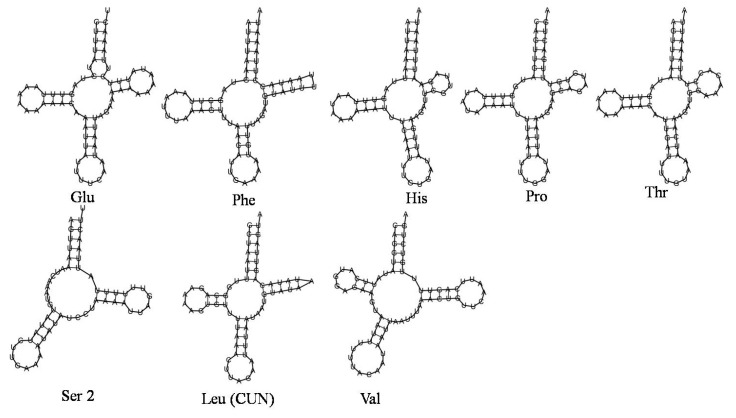
Secondary clover-leaf structures for the 23 tRNA genes of *N. lugens*. Amino acid acceptor arm, TYC arm, the anticodon arm, and dihydrouridine (DHU) arm are given in clockwise direction from the top of the tRNA.

Similar to the earlier reported insect mitochondrial genomes, *N. lugens* also has two ribosomal RNA genes, namely *rrnL* located between *trnL* and *trnV*, and *rrnS* present between the *trnV* and the CR region. The *rrnS* and *rrnL* lengths were 750 and 1020 bp, respectively, and these ranges are similar to the earlier sequenced *N. lugens* and other Delphacidae species [27,49]. The predicted secondary structure of the 23 tRNA genes is given in Figure 4. which ranges from 57 bp for *trnS* to 71 bpp for *trnK*. A characteristic clover-leaf secondary structure was observed in all of the predicted tRNAs except for *trnS* (AGN), in which the dihydrouridine arm formed a simple loop as in most insects and other metazoans [9,27,49]. Further, the *trnS* function was also previously reported to be similar to other *tRNA*s in relation to binding to ribosomes [53].

The anticodons observed in the present study are similar to those reported in other delphacid species. *N. lugens* has 7 bp of anticodon loop, 7 bp of acceptor stem, and 5 bp of anticodon stem except in lys, Ser (AGN), and Ser (UGN). Further, the loop and stem sizes of DHU and TΨC arms showed varied size differences. Additionally, 22tRNA had 37 mismatched base pairs and G-U wobble pairs. Among the 37 mismatched base pairs, a weak bond was formed by 24 G-U pairs, and the anticodon arm of *trnD*, *trnE*, and *trnL* showed an unmatched U-U base pair. Similarly, four mismatched A-A base pairs were also observed in the acceptor arm of *trnR* and the anticodon arm of *trnS*, and three unmatched A-G base pairs were observed in *trnC* and *trnW* (acceptor arm).

### 3.6. N. lugens Intergenic Spacers and Control Region

The presence of overlapping genes was observed in the *N. lugens* mitochondrial genome, similar to the other insect mitochondrial genomes [36,54]. In the present study, a total of 11 overlapping regions were observed, with the longest intergenic spacer (345 bp) found between *nad2* and *trnC*, followed by 183 bp between *trnL* and *rrnL*, 128 bp between *trnH* and *nad4*, and 111 bp between *trnM* and *nad2* (Supplementary Tables S1 and S2). The longer intergenic spacers (345 bp) reported in the present study were similar to the previous report by Zhang et al. (2013) [27] and contrasted with Lv et al. (2015) [36]. Furthermore, a significant difference exists in the gene number, intergenic spacer length, and genome size of *N. lugens* in the present study compared to the earlier reported mitochondrial genome [36]. The presence of bonus tRNA genes and intergenic spacers in the present study may be accountable for the differences in genome size reported earlier.

The control regions (CR) of the mitochondrial genomes of *N. lugens* are located between the *rrnS* and *trnI*-*trnQ*-*trnM* gene cluster (Figure 1, Supplementary Figure S1), ranging from 1304 to 1319 bp. In addition, the high A + T content (77.5%) in CR observed was also similar to previous reports [6,36]. The repeat region of the mitochondrial genome of Sample1 and Sample2 in the present study contained 15 times the repeat of unit “(G/A) ATATATATATATAAATATAT”. A similar number of repeats was also reported in *Geisha distinctissima* and *Sivaloka damnosus* [44]. However, Heteroptera species had long tandem repeats with fewer repeats as compared to the *N. ligens* mitogenome [55,56,57]. A similar number of repeats in *N. lugens* was also reported by Zhang et al. (2013) [27]. The feature for which the mitogenome of *N. lugens* was found to be highly conserved is the long poly “T” stretch (24 bp) in the A+T-rich region, which might be highly conserved in Delphacidae mitogenomes.

### 3.7. Nucleotide Diversity and Gene Evolutionary Rate

**Figure 5:**
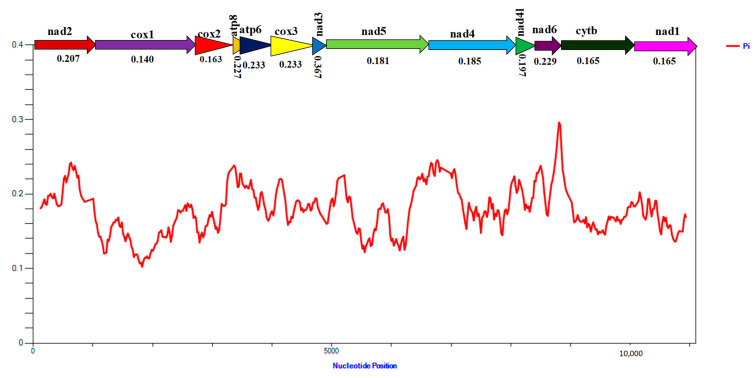
Sliding window analysis of protein-coding genes in 19 Delphacidae species from this study, including two *Nilaparvata lugens* species. The value of nucleotide diversity is represented by the red curve.

Highly variable nucleotide diversity (Pi values) was observed from the sliding window analysis, which showed significant variation between the 13 aligned PCGs of the 19 delphacid mitogenome (Figure 5). The *nad3*, *atp6*, *nad6*, and *atp8* genes displayed comparatively higher nucleotide diversities of 0.367, 0.233, 0.229, and 0.227, respectively, whereas the *cox1*, *cox2*, *nad1*, and *cytb* genes showed relatively lower nucleotide diversities of 0.140, 0.163, 0.165, and 0.165 (Figure 5). Likewise, pair wise genetic distance analysis also exhibited the same results as higher distances of 0.592, 0.326, 0.299, and 0.298 were observed for the *nad3*, *atp6*, *nad6*, and *atp8* genes, and lower distances of 0.161, 0.194, 0.197, and 0.197 were recorded for the *cox1*, *cox2*, *nad1*, and *cytb* genes, respectively.

To compute the gene evolutionary rate, the rate of synonymous substitutions (Ks, pi modifies), non-synonymous substitutions (Ka, pi modifies), and the Ka/Ks ratio were obtained for all PCGs (Table 2). The highest evolutionary rate was shown by *nad3*, followed by *nad4L*, *nad6*, and *atp6*, while the lowest rate was exhibited by *cox1*, which conforms to the fact that *cox1* is utilized as a universal barcode marker among insect species [27,36,49]. Likewise, *cytb* and *cox2* can also be used as a candidate for *N. lugens* barcode marker as they had slow evolutionary rates. However, *nad3*, *nad6*, *atp6*, and *nad4L* offer an effective marker to determine the intraspecific relationships due to their higher divergence rate, and they can expose the relationship between populations within the same insect species. The current observations are consistent with previous findings in most insects [58]. Additionally, most of the genes except *nad4L*, *nad3*, *atp6*,and *nad6* showed purifying selection based on Jukes-Cantor adjusted Ka/Ks values (<0.5).

### 3.8. Base Composition and AT/GC-Skew of Mitogenome of Delphacidae

The composition of the majority strand (J-strand) of *N. lugens* mitogenomes is A = 6846 and 6851 bp (42.6 and 42.6%), T = 5605 and 5603 bp (34.9 and 34.8%), G = 1416 and 1419 bp (8.8 and 8.8%), and C = 2195 and 2196 bp (13.7 and 13.7%) in Sample 1 and Sample 2, respectively. The nucleotide alignments of the *N. lugens* mitochondrial genomes are significantly biased toward A and T (77.5% in the present study), which was similar to the previous report (76.9%) from delphacids and other insects [27,36,49].

**Figure 6:**
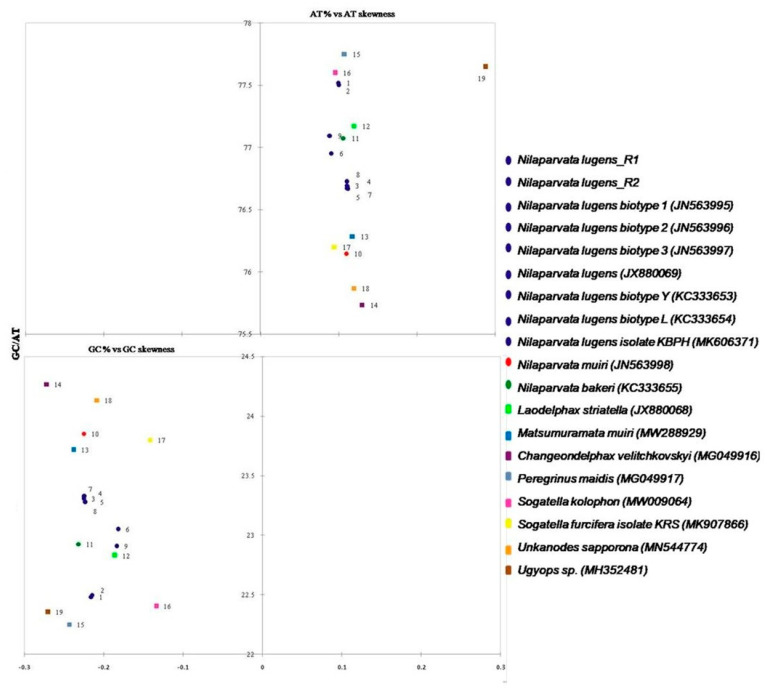
In the 19 Delphacidae species studied, including two samples of *Nilaparvata lugens*, GC % vs. GC-skew and AT % vs. AT-skew were compared. For the whole length of mitogenomes, values are determined on J-strands. The skews values are on the X-axis, while the A + T/G + C values are on the Y-axis. Color was assigned to insect species based on their taxonomic classification.

Substantial variation was observed in the base composition of the present samples compared to the mitochondrial genomes of the 19 Delphacidae samples (Figure 6): ranging from 75.7% in *Changeondelphax velitchkovskyi* (MG049916) to 77.7% in *Peregrinus maidis* (MG049917), with an average of 76.84%. In the present study, the AT skew was 0.0997 and 0.1002 in Sample 1 and Sample 2, respectively. Earlier reports suggested that the Hemipterans AT-skews vary from −0.1812 (*Trialeurodes vaporariorum*) to 0.2765 (*Lycorma delicatula*) with the *N. lugens* mitogenome AT-skew (0.0909) exhibiting similar results to our study [38]. The GC-skews of delphacids species vary from −0.1338 (*Sogatella kolophon*) to −0.2719 (*Changeondelphax velitchkovskyi*), with the *N. lugens* mitogenome displaying an analogous GC-skew (−0.2157 and−0.2149 for Sample 1 and Sample 2, respectively). These features suggest that the base compositions and base skew values of the Delphacidae mitogenomes are comparable in nature. According to a previous study, the base compositions and base skew values of the mitochondrial genomes of Hemipteran insects may be similar at low taxonomic levels (e.g., at the family level) but not at the suborder level because different taxa of Hemiptera may have been subjected to different evolutionary selection pressures [27].

### 3.9. Phylogenetic Relationship

**Figure 7:**
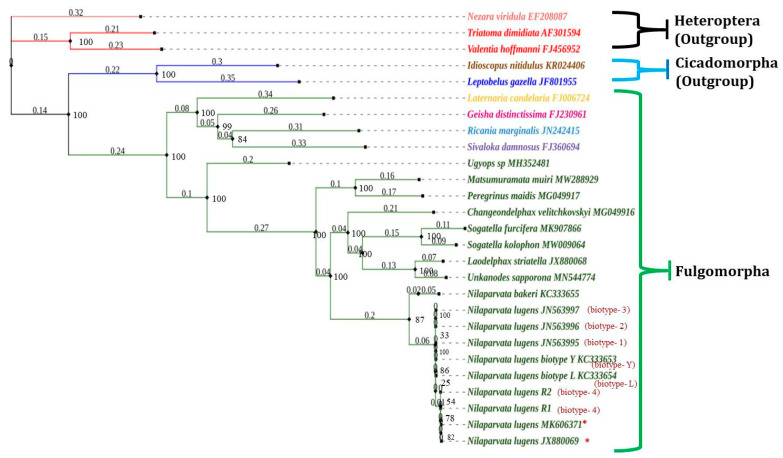
Phylogenetic tree of 19 Delphacidae species obtained from maximum likelihood on the basis of concatenated data of 13 PCGs genes. Colorations of species name and branches are given based on family which they belong. The numbers at nodes indicate branch length. Bootstrap support values are indicated at each node. Accession numbers are given for species obtained from GenBank. * Specimen biotype has not been determined.

Understanding phylogenetic relationships has been made easier because of PCGs and mitochondrial genome sequencing [37,59,60]. Earlier, the phylogenetic relationship of *N. lugens* has been inferred to a great extent with the help of partial mitochondrial gene sequences (mostly *cox1* and, in some cases, 16S rRNA genes). Whereas the current investigation employed a Maximum Likelihood technique, and a model-based evolutionary method was applied with a nucleotide sequence dataset containing 13 PCGs123 (Figure 7). Previous research suggests that when comparing the A+T content of all PCGs, the *rrnL* gene, and *rrnS* gene with the full mitochondrial genome, PCGs showed a more significant positive association (R2 = 0.9674) than the *rrnL* gene (R2 = 0.8873) and the *rrnS* gene (R2 = 0.7189). Hence, PCGs seem to better reflect the evolution of the entire mitochondrial genome than the rRNA genes. A total of 19 mitogenomes of Delphacidae (including nine *N. lugens*) were used in our phylogenetic analysis, along with two species from Cicadamorpha and three species from Heteroptera. The results indicated that Cicadamorpha and Heteroptera species clearly formed outgroups with all other selected Fulgoromorpha grouped together. The phylogenetic analysis of Fulgoromorpha based on ML yielded two topologies (Figure 7) when rooted with *Ugyops* sp in one clade; the remaining species form another clade (Delphacini tribe), with *P. maidis* and *M. muiri* being sisters to the remaining species. The present result of *P. maidis* as a sister to the remaining Delphacini was similar to previous reports [61]. Further, the conformation of the genus *Nilaparvata* forms a separate subclade from the other genera(*Sogatella*, *Laodelphax*, *Changeondelphax*, and *Unkanodes*) belonging to Delphacidae. The confirmation of the order of the clade comprising three rice planthoppers (*N. lugens*, *S. furcifera*, and *L. striatelus*) was *L. striatelus* + (*S. furcifera + S. kolophon*) + (*N. lugens* + *N. bakeri* + *N. muiri*). Additionally, the relationship between thebiotypes of *N. lugens* was recovered as the present experiment samples (biotype-4) were separated from the earlier reported biotypes viz., biotype-1 (JN563995), biotype-2 (JN563996), biotype-3 (JN563997), biotype-Y (KC333653), and biotype-L (KC333654). Earlier reports also support our phylogeny claim [26,35,37,60].

## 4. Conclusions

The present study documents the first mitogenome of *N. lugens* biotype-4 from India, which contains 37 characteristic metazoan mitochondrial genes and retains the organizational structure of the many other Delphacidae mitogenomes along with additional repeats of *trnC*. *N. lugens* mitochondrial genome rearrangement occurred in the present study, suggesting that such rearrangement was conserved in Delphacidae. Compared to other hemipterans and insects, *N. lugens* have an extremely short length of *atp8* genes with one additional repeat of *trnC*, for which functional characterization needs to be ascertained in the future for their essential role in *N. lugens*. The present study’s sequenced mitogenome dataset is valuable information that contributesto future phylogenetic and evolutionary studies. Protein Coding Genes seem to better reflect the evolution of the entire mitochondrial genome than *rRNA* genes. Hence, phylogeny reconstruction on the basis of protein-coding genes in more taxa of delphacids is required for a good understanding of the evolution of Delphacidae.

## Figures and Tables

**Table 1:** Codon usage and relative synonymous codon usage within *Nilaparvata lugens* mitogenome.

U				C				A				G			
Codon		Count	RSCU	Codon		Count	RSCU	Codon		Count	RSCU	Codon		Count	RSCU
UUU	Phe	25.5	1.70	UCU	Ser	8.5	2.27	UAU	Tyr	9.6	1.66	UGU	Cys	3.0	1.86
UUC	Phe	04.5	0.30	UCC	Ser	2.5	0.66	UAC	Tyr	2.0	0.34	UGC	Cys	0.2	0.14
UUA	Leu	25.7	3.88	UCA	Ser	7.3	1.96	UAA	Stop	0.0	0.00	UGA	Trp	5.2	1.72
UUG	Leu	03.5	0.52	UCG	Ser	0.5	0.12	UAG	Stop	0.0	0.00	UGG	Trp	0.8	0.28
CUU	Leu	04.8	0.73	CCU	Pro	3.4	1.36	CAU	His	3.5	1.41	CGU	Arg	1.1	1.30
CUC	Leu	00.8	0.12	CCC	Pro	2.9	1.18	CAC	His	1.5	0.59	CGC	Arg	0.1	0.09
CUA	Leu	04.5	0.67	CCA	Pro	3.4	1.36	CAA	Gln	3.1	1.67	CGA	Arg	1.8	2.14
CUG	Leu	00.5	0.07	CCG	Pro	0.2	0.09	CAG	Gln	0.6	0.33	CGG	Arg	0.4	0.47
AUU	Ile	26.1	1.76	ACU	Thr	4.5	1.62	AAU	Asn	11.2	1.66	AGU	Ser	3.9	1.05
AUC	Ile	03.5	0.24	ACC	Thr	2.2	0.81	AAC	Asn	2.3	0.34	AGC	Ser	0.5	0.14
AUA	Met	16.5	1.72	ACA	Thr	4.1	1.48	AAA	Lys	8.9	1.72	AGA	Ser	5.9	1.59
AUG	Met	02.7	0.28	ACG	Thr	0.2	0.08	AAG	Lys	1.5	0.28	AGG	Ser	0.8	0.21
GUU	Val	04.8	1.84	GCU	Ala	2.8	1.89	GAU	Asp	3.3	1.48	GGU	Gly	4.8	1.56
GUC	Val	00.6	0.23	GCC	Ala	1.0	0.68	GAC	Asp	1.2	0.52	GGC	Gly	0.5	0.15
GUA	Val	04.5	1.69	GCA	Ala	2.0	1.37	GAA	Glu	5.5	1.80	GGA	Gly	5.1	1.63
GUG	Val	00.6	0.23	GCG	Ala	0.1	0.05	GAG	Glu	0.6	0.20	GGG	Gly	2.1	0.67

**Table 2:** Nucleotide substitution measure in each PCG of mitogenome of 19 Delphacidae species including two samples of *Nilaparvata lugens* used from this study.

Protein-Coding Genes	Rates of Non-Synonymous Substitutions (Ka)	Rates of Synonymous Substitutions (Ks)	Ka/Ks Ratio	Rates of Non-Synonymous Substitutions Jukes-Cantor Adjusted J(Ka)	Rates of Synonymous Substitutions Jukes-Cantor Adjusted J(Ks)	JKa/JKs Ratio
*nad2*	0.15659	0.40221	0.38932	0.18751	0.72155	0.25987
*cox1*	0.04354	0.46370	0.09390	0.04562	0.98025	0.04654
*cox2*	0.09142	0.43730	0.20906	0.10113	0.86819	0.11648
*atp8*	0.18724	0.39761	0.47091	0.23575	0.73457	0.32094
*atp6*	0.18595	0.40582	0.45821	0.25526	0.71953	0.35476
*cox3*	0.11981	0.44117	0.27157	0.13657	0.85826	0.15912
*nad3*	0.32060	0.54762	0.58544	0.37831	0.96928	0.39029
*nad6*	0.19594	0.36638	0.53480	0.24547	0.59248	0.41431
*cytB*	0.08143	0.47195	0.17254	0.08905	1.00887	0.08827
*nad1*	0.10299	0.39094	0.26344	0.11561	0.65987	0.17520
*nad4L*	0.16987	0.29200	0.58175	0.20935	0.40849	0.51250
*nad4*	0.14236	0.33225	0.42847	0.16642	0.49613	0.33544
*nad5*	0.14155	0.32656	0.43346	0.16546	0.48086	0.34409

## Data Availability

All the data generated in this work was provided in the article.

## Supplementary Materials

The following supporting information can be downloaded at: https://www.mdpi.com/article/10.3390/life12091289/s1, Table S1: Summary table for characteristics of the complete mitogenome of *Nilaparvata lugens_1* (NCBI Accession Number: OK585089). Table S2: Summary table for characteristics of the complete mitogenome of *Nilaparvata lugens_2* (NCBI Accession Number: OM372598). Figure S1: Mitochondrial genome map of *Nilaparvata lugens* sample 2. From outer to inner, the 1st circle shows the gene map (PCGs, rRNA, tRNAs, and CR) and tRNA genes are abbreviated by one letter symbols according to the IUPAC-IUB single-letter amino acid codes. The 2nd circle shows the GC content and the 3rd shows GC skew calculated as (G − C)/(G + C). GC content and GC skew are plotted as the deviation from the average value of the entire sequence.
