# Supplementary material for: Genome Organization and Comparative Evolutionary Mitochondriomics of Brown Planthopper, Nilaparvata lugens Biotype 4 Using Next Generation Sequencing (NGS)
Source: Life (Basel). 2022 Aug 23;12(9):1289. doi: 10.3390/life12091289 (PMC9506247; doi:10.3390/life12091289)
Supplement: Supplementary file 1 [file life-12-01289-s001.zip › life-1837705-SI.pdf]

Supplementary Table S1: Summary table for characteristics of the complete mitogenome of *Nilaparvata lugens_1* (NCBI Accession Number: OK585089)

Gene	*Location*	*Direction*	*Size*	*Intergenic Spacers*	*Anticodon*	*Start codon*	*Stop codon*
tRNA-Ile	*1-69*	+	*69*		*atc*		
tRNA-Gln	*73-139*	-	*67*	*3*	*caa*		
tRNA-Met	*139-202*	+	*64*	*-1*	*atg*		
NADH dehydrogenase subunit 2	*224-1045*	+	*822*	*21*		*ata*	*tta*
tRNA-Cys	*1157-1219*	-	*63*	*111*	*tgc*		
tRNA-Cys	*1565-1627*	-	*63*	*345*	*tgc*		
tRNA-Trp	*1638-1702*	+	*65*	*10*	*tga*		
tRNA-Tyr	*1712-1774*	-	*63*	*9*	*tac*		
cytochrome c-oxidase subunit 1	*1788-3302*	+	*1515*	*13*		*atg*	*taa*
tRNA-Leu	*3322-3387*	+	*66*	*19*	*tta*		
cytochrome c-oxidase subunit 2	*3388-4041*	+	*654*	*0*		*att*	*tga*
tRNA-Lys	*4062-4132*	+	*71*	*20*	*aaa*		
tRNA-Asp	*4133-4196*	+	*64*	*0*	*gac*		
ATPase subunit 8	*4197-4289*	+	*93*	*0*		*att*	*t*
ATPase subunit 6	*4292-4939*	+	*648*	*2*		*ata*	*taa*
cytochrome c-oxidase subunit 3	*4947-5717*	+	*771*	*7*		*atg*	*tga*
tRNA-Gly	*5725-5786*	+	*62*	*7*	*gga*		
NADH dehydrogenase subunit 3	*5793-6134*	+	*342*	*6*		*ata*	*t*
tRNA-Ala	*6139-6199*	+	*61*	*4*	*gca*		
tRNA-Arg	*6204-6263*	+	*60*	*4*	*cga*		
tRNA-Asn	*6263-6326*	+	*64*	*-1*	*aac*		
tRNA-Ser	*6326-6384*	+	*59*	*-1*	*aga*		
tRNA-Glu	*6384-6448*	+	*65*	*-1*	*gaa*		
tRNA-Phe	*6449-6514*	-	*66*	*0*	*ttc*		
NADH dehydrogenase subunit 5	*6528-8144*	-	*1617*	*13*		*atg*	*tt*
tRNA-His	*8190-8250*	-	*61*	*45*	*cac*		
NADH dehydrogenase subunit 4	*8274-9437*	-	*1164*	*23*		*att*	*taa*
NADH dehydrogenase subunit 4L	*9566-9829*	-	*264*	*128*		*atg*	*taa*
NADH dehydrogenase subunit 6	*9884-10357*	+	*474*	*54*		*att*	*t*
tRNA-Pro	*10448-10509*	-	*62*	*90*	*cca*		
tRNA-Thr	*10510-10572*	+	*63*	*0*	*aca*		
apocytochrome b	*10580-11653*	+	*1074*	*7*		*atg*	*t*
tRNA-Ser	*11682-11738*	+	*57*	*28*	*tca*		
NADH dehydrogenase subunit 1	*11768-12673*	-	*906*	*29*		*atg*	*t*

tRNA-Leu	*12675-12736*	-	*62*	*1*	*Cta*		
16S ribosomal RNA	*12753-13772*	-	*1020*	*16*			
tRNA-Val	*13956-14024*	-	*69*	*183*	*Gta*		
12S ribosomal RNA	*14019-14768*	-	*750*	*-6*			
Probable D-loop	*14769-16072*		*1304*	*0*			

Supplementary Table S2: Summary table for characteristics of the complete mitogenome of *Nilaparvata lugens_2* (NCBI Accession Number: OM372598**)**

Gene	***Location***	***Direction***	***Size***	***Intergenic Spacers***	***Anticodon***	***Start codon***	***Stop codon***
tRNA-Ile	*1-69*	+	*69*		*atc*		
tRNA-Gln	*73-139*	-	*67*	*3*	*caa*		
tRNA-Met	*139-202*	+	*64*	*-1*	*atg*		
NADH dehydrogenase subunit 2	*224-1045*	+	*822*	*21*		*ata*	*tta*
tRNA-Cys	*1157-1219*	-	*63*	*111*	*tgc*		
tRNA-Cys	*1565-1626*	-	*63*	*345*	*tgc*		
tRNA-Trp	*1637-1701*	+	*65*	*10*	*tga*		
tRNA-Tyr	*1711-1773*	-	*63*	*9*	*tac*		
cytochrome c-oxidase subunit 1	*1787-3301*	+	*1515*	*13*		*atg*	*taa*
tRNA-Leu	*3321-3386*	+	*66*	*19*	*tta*		
cytochrome c-oxidase subunit 2	*3387-4040*	+	*654*	*0*		*att*	*tga*
tRNA-Lys	*4061-4131*	+	*71*	*20*	*aaa*		
tRNA-Asp	*4132-4195*	+	*64*	*0*	*gac*		
ATPase subunit 8	*4196-4288*	+	*93*	*0*		*att*	*t*
ATPase subunit 6	*4291-4938*	+	*648*	*2*		*ata*	*taa*
cytochrome c-oxidase subunit 3	*4946-5716*	+	*771*	*7*		*atg*	*tga*
tRNA-Gly	*5724-5785*	+	*62*	*7*	*gga*		
NADH dehydrogenase subunit 3	*5792-6133*	+	*342*	*6*		*ata*	*t*
tRNA-Ala	*6138-6198*	+	*61*	*4*	*gca*		
tRNA-Arg	*6203-6262*	+	*60*	*4*	*cga*		
tRNA-Asn	*6262-6325*	+	*64*	*-1*	*aac*		
tRNA-Ser	*6325-6383*	+	*59*	*-1*	*aga*		
tRNA-Glu	*6383-6447*	+	*65*	*-1*	*gaa*		
tRNA-Phe	*6448-6513*	-	*66*	*0*	*ttc*		
NADH dehydrogenase subunit 5	*6527-8143*	-	*1617*	*13*		*atg*	*tt*
tRNA-His	*8189-8249*	-	*61*	*45*	*cac*		
NADH dehydrogenase subunit 4	*8273-9436*	-	*1164*	*23*		*att*	*taa*
NADH dehydrogenase	*9565-9828*	-	*264*	*128*		*atg*	*taa*

subunit 4L							
NADH dehydrogenase subunit 6	*9883-10356*	+	*474*	*54*		*att*	*t*
tRNA-Pro	*10442-10503*	-	*62*	*85*	*cca*		
tRNA-Thr	*10504-10566*	+	*63*	*0*	*aca*		
apocytochrome b	*10574-11647*	+	*1074*	*7*		*atg*	*t*
tRNA-Ser	*11676-11732*	+	*57*	*28*	*aga*		
NADH dehydrogenase subunit 1	*11762-12667*	-	*906*	*29*		*atg*	*t*
tRNA-Leu	*12669-12730*	-	*62*	*1*	*tta*		
16S ribosomal RNA	*12747-13766*	-	*1020*	*16*			
tRNA-Val	*13950-14018*	-	*69*	*183*	*gta*		
12S ribosomal RNA	*14013-14762*	-	*750*	*-6*			
Probable D-loop	*14763-16081*		*1319*	*0*			

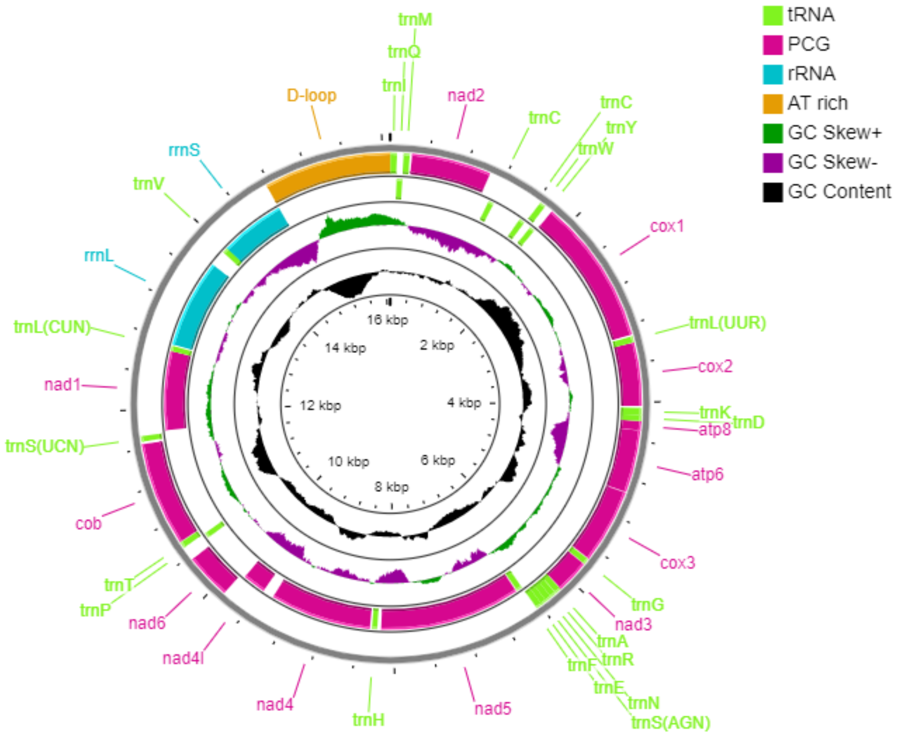

Supplementary Figure S1. Mitochondrial genome map of *Nilaparvata lugens* sample-2. From outer to inner, the 1st circle shows the gene map (PCGs, rRNA, tRNAs & CR) and tRNA genes are abbreviated by one letter symbols according to the IUPAC-IUB single-letter amino acid codes. The 2ndcircle shows the GC content and the 3rdshows GC skew calculated as (G-C)/(G+C). GC content and GC skew are plotted as the deviation from the average value of the entire sequence.
